# Chronic Inflammation and Angiogenic Signaling Axis Impairs Differentiation of Dental-Pulp Stem Cells

**DOI:** 10.1371/journal.pone.0113419

**Published:** 2014-11-26

**Authors:** Michael Boyle, Crystal Chun, Chelsee Strojny, Raghuvaran Narayanan, Amelia Bartholomew, Premanand Sundivakkam, Satish Alapati

**Affiliations:** 1 Department of Endodontics, College of Dentistry, University of Illinois at Chicago, Chicago, Illinois, United States of America; 2 Department of Surgery, College of Medicine, University of Illinois at Chicago, Chicago, Illinois, United States of America; University of Minnesota Medical School, United States of America

## Abstract

Dental-pulp tissue is often exposed to inflammatory injury. Sequested growth factors or angiogenic signaling proteins that are released following inflammatory injury play a pivotal role in the formation of reparative dentin. While limited or moderate angiogenesis may be helpful for dental pulp maintenance, the induction of significant level of angiogenesis is probably highly detrimental. Hitherto, several studies have addressed the effects of proinflammatory stimuli on the survival and differentiation of dental-pulp stem cells (DPSC), *in vitro*. However, the mechanisms communal to the inflammatory and angiogenic signaling involved in DPSC survival and differentiation remain unknown. Our studies observed that short-term exposure to TNF-α (6 and 12 hours [hrs]) induced apoptosis with an upregulation of VEGF expression and NF-κB signaling. However, long-term (chronic) exposure (14 days) to TNF-α resulted in an increased proliferation with a concomitant shortening of the telomere length. Interestingly, DPSC pretreated with Nemo binding domain (NBD) peptide (a cell permeable NF-κB inhibitor) significantly ameliorated TNF-α- and/or VEGF-induced proliferation and the shortening of telomere length. NBD peptide pretreatment significantly improved TNF-α-induced downregulation of proteins essential for differentiation, such as bone morphogenic proteins (BMP)-1 & 2, BMP receptor isoforms-1&2, trasnforming growth factor (TGF), osteoactivin and osteocalcin. Additionally, inhibition of NF-κB signaling markedly increased the mineralization potential, a process abrogated by chronic exposure to TNF-α. Thus, our studies demonstrated that chronic inflammation mediates telomere shortening via NF-κB signaling in human DPSC. Resultant chromosomal instability leads to an emergence of increased proliferation of DPSC, while negatively regulating the differentiation of DPSC, *in vitro*.

## Introduction

Dental-pulp stem cells (DPSC) contribute to dentinogenesis, a process required for mineralization. Subsequently, the elaboration of collagenous extracellular matrix (ECM) actively promotes the dental-pulp regeneration and maintains the integrity of dental-pulp tissue [1], [2]. Hence, DPSC were considered an ideal tool to regain lost dental tissues and to re-engineer the root canal system [3]. DPSC differentiate not only as osteoblasts and odontoblasts, but also into several different cell types including adipocytes, neurons, chondrocytes, mesenchymal stem cells (MSC) and endothelial cells [4]–[6].

Dental caries is the most prevalent infectious disease among children and adults [7]. Dental caries or trauma can result in an inflammatory response, characterized by an accumulation of inflammatory cells, which release host proinflammatory cytokines, including tumor necrosis factor-α (TNF-α) and interleukins [8]–[11]. Hence, TNF-α has been documented as a marker of early inflammation and plays a key role in the inflammatory response [12]. TNF-α was also shown to affect osteoclastogenesis and bone formation [13]. Additionally, prolonged exposure to inflammatory environment also is evident to lead to chronic hypoxia, an ensuing cause for altered metabolic shift-oriented cellular energy status, angiogenic switch, dilated blood vessels with an associated increase in blood flow changes, vasodilation and vascular permeability, chronic hypoxia, increased pulpal pressure and neuronal activity, associated with an intense pain [14]–[16]. Hitherto, studies have postulated an increased apoptotic signaling with a compromised longevity of DPSC upon short term exposure to inflammatory signaling [17]. However, this situation contradicts to the scenario exhibited in the diseased pulpal tissue, where weak and hyperproliferative pulp cells prevail with a diminished mineralization potential. However, the mechanisms contributing to the prolonged exposure to inflammation remain unclear.

Several lines of studies have shown the crucial role of nuclear factor-kappa B (NF-κB) [18]–[20] in inflammation-induced downstream signaling mechanisms. In the unstimulated condition, NF-κB is retained in the cytoplasm in the most common form (p65 and p65/RelA heterodimers) by the inhibitory protein IκBα. Upon stimulation by TNF-α or other inflammatory stimuli, IKK-α and IKK-β are activated following IKK-γ ubiquitination by undetermined mechanisms [18], [21]. The activated IKK complex then phosphorylates IκB-α at the serine residues in the N-terminal region. The phosphorylated IκB-α is subsequently ubiquitinated and degraded by the 26S proteasome machinery. The degradation of IκB-α then activates NF-κB signaling [18], [22], [23]. In this study, to understand the role of inflammation and host response, we examined whether prolonged exposure to TNF-α activates the NF-κB signaling pathway in DPSC.

Angiogenesis, the formation of new blood vessels from pre-existing ones, plays a crucial role in a variety of physiological and pathological processes, such as chronic inflammation, wound healing, and tissue regeneration. In dental-pulp tissue, vascular angiogenesis is an indeterminant phase for physiological tooth development and for healing pulpal injury [24]–[26]. Studies have shown that the inflamed tissues enhance the expression of mitogenic factors such as vascular endothelial growth factor (VEGF), fibroblast growth factor (FGF), and platelet-derived growth factor (PDGF) in human pulp and gingival fibroblasts [25], [27], [28]. These factors were demonstrated to contribute to the destruction of pulpal and periapical tissues with the expansion of the vascular network coincident to progression of the inflammation. Furthermore, studies have shown that the mitogenic factors, especially VEGF promote the proliferation and differentiation potential of DPSC [29], [30]. These findings cumulatively suggest that upregulation of angiogenic signaling during inflammatory processes significantly contributes to the pathogenesis associated with DPSC survival and differentiation into mature odonotoblast-like cells. Therefore, when studying the effects of inflammation, it is highly imperative to investigate the communal effects of inflammatory mediators and angiogenic molecules in arbitrating DPSC differentiation and proliferation. Since, inflammatory cytokines in conjunction with angiogenic signaling are essential for reparative dentinogenesis, the aim of this study was to examine the effect of TNF-α and angiogenic factors in mediating the proliferation and differentiation potentials of DPSC.

## Materials and Methods

### Human DPSC Isolation and Culture

Human DPSC (generously donated by Dr. Songtao Shi, University of Southern California) were collected from the third molars of patients undergoing extraction for orthodontic or therapeutic reasons [31]. Written informed consent of patients was obtained via their guardians. This study was approved by the medical ethical committee of Office of the Protection of Research Subjects (OPRS), University of Illinois at Chicago. Dental pulp tissue was obtained with forceps after mechanically fracturing the teeth with surgical chisels. DPSC were isolated from the pulp tissue and the single cell suspensions were cultured in αMEM (Gibco), supplemented with 20% FBS (Hyclone, UT, USA), 1% Antibiotic-antimycotic (Gibco). Odontogenic medium was supplemented with 100 µM/ml ascorbic acid, 2 mM β-glycerophosphate, and 10 mM dexamethasone. DPSC were incubated at 37°C with 5% CO_2_. DPSC between 3^rd^ and 5^th^ passages were used throughout the study. Treatment of TNF-α for 4 and 6 hrs were considered as short term; whereas treatment for 14 days was termed as long term exposure. DPSC were cultured in 3% serum containing media for all the experiments performed in this study.

### Real Time PCR Analysis

Total RNA from DPSC was extracted using TRIzol reagent. Reverse transcription (RT) was performed using oligo (dT) primers and superscript RT (Invitrogen) following the manufacturer's instructions. Human p65, BCL2, Survivin, BMP, BMPR, TGF-β1, TGF-β2, VEGF, EGF, FGF-1, FGF-2, osteocalcin, osteoactivin, RUNX2, and GAPDH were amplified using the primer sets (Table 1). RT product (2 µl) was amplified in a 10-µl volume with iQ™ SYBR Green supermix (Bio-Rad laboratories). Reactions were performed using ABI PRISM 7000 Sequence Detection System (Applied Biosystems, CA).

**Table 1:** The Human Primer Sequences used for Real-Time PCR.

Human Gene	Primers
GAPDH	For: GGCATCCACTGTGGTCATGAG
	Rev: TGCACCACCAACTGCTTAGC
VEGF	For: CAAAAACGAAAGCGCAAGAAA
	Rev: GCGGGCACCAACGTACAC
EGF	For: GGGCATGACTAATTCCCACTGA
	Rev: GCCCAATCCTAGACGGCAAC
FGF-1	For: AAGGGCTTTTATACGGCTCG
	Rev: CCCACAAACCAGTTCTTCTCC
FGF-2	For: GGCTTCTTCCTGCGCATCCA
	Rev: GCTCTTAGCAGACATTGGAAGA
BCL2	For: CTGCACCTGACGCCCTTCACC
	Rev: CACATGACCCCACCGAACTCAAAGA
Survivin	For: GCCCAGTGTTTCTTCTGCTT
	Rev: CCGGACGAATGCTTTTTATG
p65	For: AGCACCATCAACTATGATGAGTTTC
	Rev: GAGTTATAGCCTCAGGGTACTCCAT
BMP	For: TCAAGCCAAACACAAACAGC
	Rev: AGCCACAATCCAGTCATTCC
BMPR	For: AGCTACGCCGGACAATAGAA
	Rev: CTATGACAACAGGGGGCAGT
TGF-β1	For: CCCAGCATCTGCAAAGCTC
	Rev: GTCAATGTACAGCTGCCGCA
TGF-β2	For: TGCCGCCCTTCTTCCCCTC
	Rev: GGAGCACAAGCTGCCCACTGA
Osteocalcin	For: TGC TTG AGG AGG AAG TTC AC
	Rev: AGG TCA CTG CCC ACA GAG TA
Osteoactivin	For: CTCCTGAGAGTCTGACAAAGCCTT
	Rev: GCTGTGACATCCATTACTTGC
RUNX2	For: CCTGAACTCTGCACCAAGTC
	Rev: GAGGTGGCAGTGTCATCATC

### CFSE Staining and Flow Cytometry Analysis

After appropriate treatment conditions and at the respective time points, DPSC were labeled for 10 min at 37°C with 2 µM CFSE (Invitrogen) in Dulbecco's PBS (D-PBS; Invitrogen) supplemented with 3% FBS (Invitrogen). The same volume of ice-cold D-PBS with 10% FBS was then added to stop the reaction. After washing with Mg^2+^/Ca^2+^-free PBS, 1×10^5^ CFSE-labeled DPSC were the subjected to flow cytometry analysis. Each single division was determined as follows: a gate for zero division was set on the CFSE peak of the undivided naive cells, and subsequent divisions were determined according to reduced fluorescence intensity of peaks in respective histograms. The percentage of cells in different generations was plotted, accordingly. Cells treated with concanavlin A (Sigma-Aldrich, St. Louis, MO, USA) were used as a positive control, whereas media alone served as a negative control.

### Flow Cytometry Analysis

For flow cytometry, 1×10^5^ cells were incubated with FITC-conjugated primary mAbs against PE-conjugated CD29, CD105 and APC-CD45 and CD31 (BD Biosciences) at 4°C for 30 minutes and then washed twice with PBS containing 0.1% bovine serum albumin. The side population (SP) cells were stained with antibody Bcrp1/ABC-G2-PE (R&D Systems Inc.). The expression of intracellular markers was examined by indirect immunostaining. Cells were fixed with 4% (w/v) PFA for 5 minutes and permeabilized with 0.1% (v/v) Triton X-100 in PBS for 5 minutes. The secondary Abs were anti-mouse IgG, anti-rabbit IgG, and anti–guinea pig IgG-conjugated with Alexa Fluor 448 (Invitrogen), used at 1∶1,000. Cell fluorescence was evaluated by flow cytometry using a FACSCalibur (BD Biosciences). Three samples from each experiment were analyzed.

### BrdU Incorporation Assay

For proliferation studies, DPSC were cultured to approximately 50% confluence in 96-well plates (BD Bioscience). At the end of the treatment period, cells were starved overnight in low-serum media, followed by an 18-hour pulse with 10 µM 5-bromo-2′-deoxyuridine (BrdU) in EB-CM from different time points as well as control media. After the 18-hour pulse, cells were rinsed with PBS and fixed in 70% ethanol with 2 M HCl for 10 minutes at room temperature, then rinsed in PBS at least three times. The cell lysates were then measured at excitation: 450 nm and emission: 595 nm using ELISA plate reader (Thermo Scientific, USA).

### MTT Assay

DPSC cultured on 96-well plate at concentration 1×10^3^ cells/well were subjected to appropriate treatment conditions, while grown in ondonto-induction medium. The formation of formazan products was measured spectrophotometrically, at appropriate time periods, using methylthiazolyldiphenyl-tetrazolium bromide (MTT) assay kit. The culture medium was replaced with 5 mg/mL MTT solution in PBS and the plates were incubated for 6 h at 37°C. The precipitate was extracted with DMSO and optical density was measured at wavelength 550 nm.

### Alizarin Red Staining

DPSC seeded onto 12-well plates (1×10^4^ cells per well) were subjected to alizarin red (ALR) staining at day 14. Briefly, the cells were fixed in 4% paraformaldehyde for 20 min, then stained using alizarin red (Sigma-Aldrich). The phase contrast images were then captured for analysis using EVOS FL Cell Imaging System (Life Technologies).

### Alkaline Phosphatase Activity

DPSC were grown in odonto-induction media for 14 days, at 37°C. Cells were then fixed with 4% paraformaldehyde and fluorescence alkaline phospahatase (ALP) detection assay was performed according to the manufacturer's instruction (Sigma-Aldrich).

### Western Blot

DPSC lysates were resolved by SDS-polyacrylamide gel electrophoresis on a 10% separating gel under reducing conditions and transferred to Duralose membrane. Membranes were blocked with (5% dry milk in 10 mM Tris-HCl, pH 7.5, 150 mM NaCl, and 0.05% Tween 20) for 1 h. Membranes were incubated with indicated primary antibody (diluted in blocking buffer) overnight. After three washes, membranes were incubated with horseradish peroxidase-conjugated secondary antibody. Protein bands were detected by enhanced chemiluminescence.

### Telomere Length

Average telomere length was measured from total genomic DNA of human DPSC by using a sequence-independent multiplex qPCR technique utilizing a SYBR Green master mix with 0.625 U AmpliTaq Gold 360 DNA polymerase (Life Technologies). Each reaction included 10 µL 2× SYBR Green mix (Bio-Rad), 0.5 µL each of 10 µM forward and reverse primers, 4 µL water and 5 µL genomic DNA (10 ng/µL) to yield a 20-µL reaction. DNA samples were placed in adjacent three wells of a 96-well plate for telomere primers and reference gene primers, respectively. A Bio-Rad thermocycler (CFX96 system) was used with reaction conditions of 95°C for 10 min followed by 40 cycles of data collection at 95°C for 15 s, 60°C anneal for 30 s and 72°C extend for 30 s along with 80 cycles of melting curve from 60°C to 95°C. CFX manager software was used to generate standard curves and Ct values for telomere signals and reference gene signals.

### Statistical Analysis

Comparisons were made with a two-tailed Student's *t* test. Experimental values were reported as mean ± S.E. Differences in mean values between two or more groups were determined by one-way analysis of variance. A *p* value<0.05 was considered statistically significant.

## Results

### Short-Term Exposure of TNF-α Induces Apoptosis via NF-κB Signaling Pathway in DPSC

**Figure 1:**
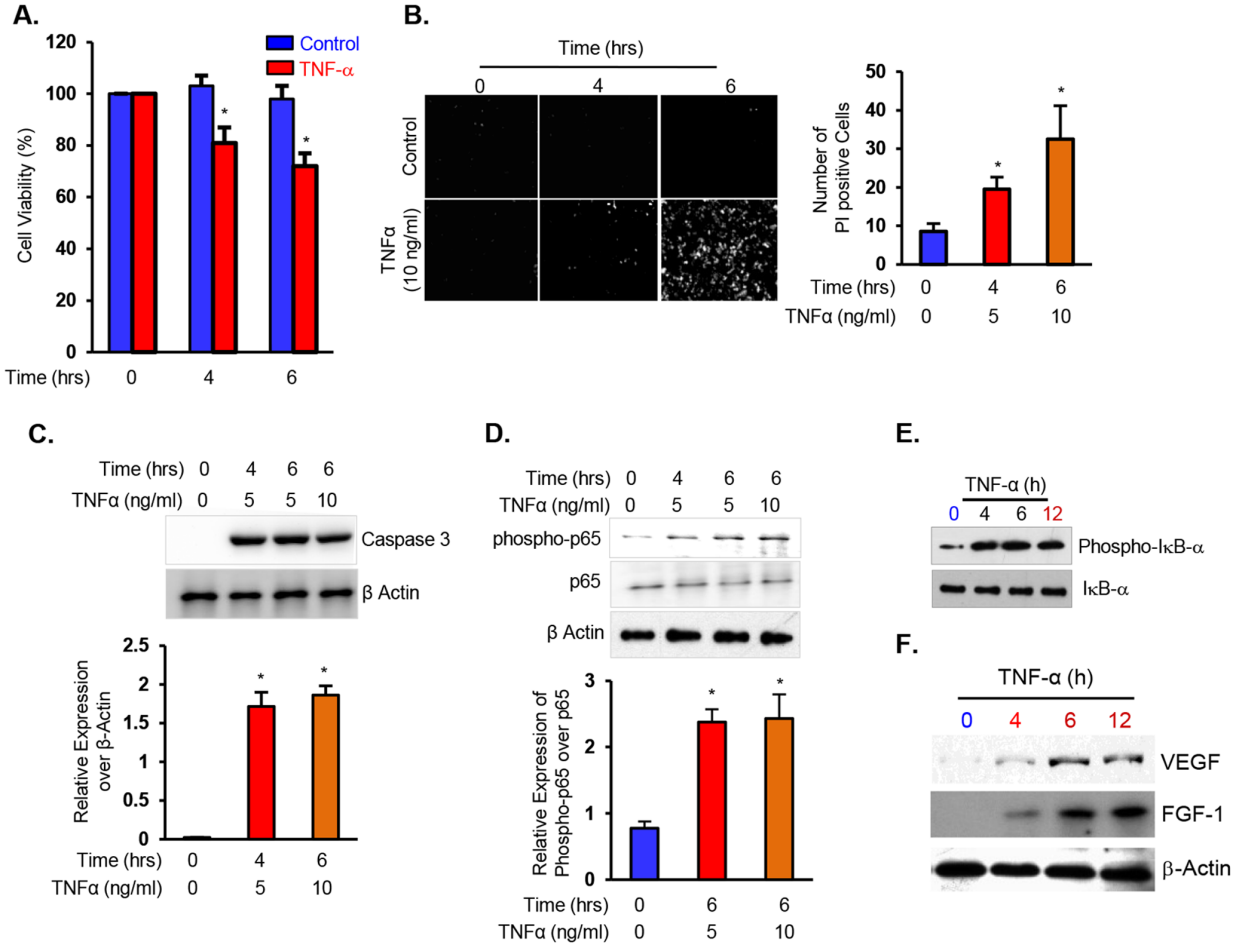
Short-term exposure to TNF-α induces apoptosis via NF-κB signaling with an associated increase in VEGF expression in DPSC. (**A**) DPSC were cultured in 3% serum containing medium for 0, 4, and 6 hrs in the absence or presence of TNF-α, and cell viability was assessed using MTT assay. (**B**) DPSC grown to approximately 80% confluence was immunostained for propidium iodide in the absence or presence of TNF-α at 0, 4, and 6 hrs. (**C**) Representative Western blot data showing an increase in Caspase 3 expression upon challenging with varying concentrations of TNF-α (0, 5, and 10 ng/ml) at 4 and 6 hrs. (**D**) Western blot analysis of extracts from DPSC showing an increase in the expression of phospho-p65 (upper panel) upon challenging with TNF-α for varying time points. (**E**) Western blot analysis showing the expression levels of phospho-I-κB-α and I-κB-α. (**F**) VEGF and FGF levels in DPSC challenged with TNF-α for 0, 4, 6, and 12 hrs in 3% serum containing medium. Note an increase in the expression of VEGF and FGF following TNF-α treatment at 6 and 12 hrs. The data shown are Mean ± SD. *p<0.05.

To examine the reparative response of DPSC to short-term exposure with proinflammatory stimuli, we challenged cells with TNF-α for varying time points (0, 4, and 6 hrs) in 3% serum containing medium. As shown in Fig. 1A, we observed a significant decrease in the number of viable DPSC at 4 and 6 hrs, as determined using MTT assay. Additionally, we observed an increase in the propidium iodide positive cells, representing the number of apoptotic cells, (Fig. 1B) and an increase in the levels of caspase-3 expression (Fig. 1C) which confirm our findings that short-term exposure of TNF-α induce cell death, *in vitro*.

To address whether TNF-α-induced apoptosis occurs via NF-κB signaling pathway, we examined the activation of p65 using Western blot analysis. Interestingly, we observed an increase in the levels of phospho-p65 (∼1.5-fold) upon TNF-α induction at 4 and 6 hrs (Fig. 1D). Next, we investigated the role of TNF-α in mediating the activation of IκB-α, on the basis of the proposal that NF-κB signaling is required for IκB-α expression and that IκB-α in a negative feedback manner inhibits NF-κB activation. We observed that the basal expression levels of phospho-IκB-α was markedly lower in DPSC; however TNF-α treatment for varying time points (0, 4, 6, and 12 hrs) significantly increased the levels of phospho-IκB-α (Fig. 1E). To determine whether TNF-α influences the expression of VEGF, an angiogenic signaling protein, DPSC treated with TNF-α for 0, 4, 6 and 12 hrs were subjected to western blot analysis. As shown in Fig. 1F, we observed a significant increase in the levels of VEGF at 6 and 12 hrs, suggesting that that short-term exposure of TNF-α upregulated angiogenic signaling concomitant with the apoptosis via NF-κB signaling pathway.

### Prolonged Exposure of TNF-α Induces Proliferation and Phenotypic Alterations of DPSC, *In Vitro*

**Figure 2:**
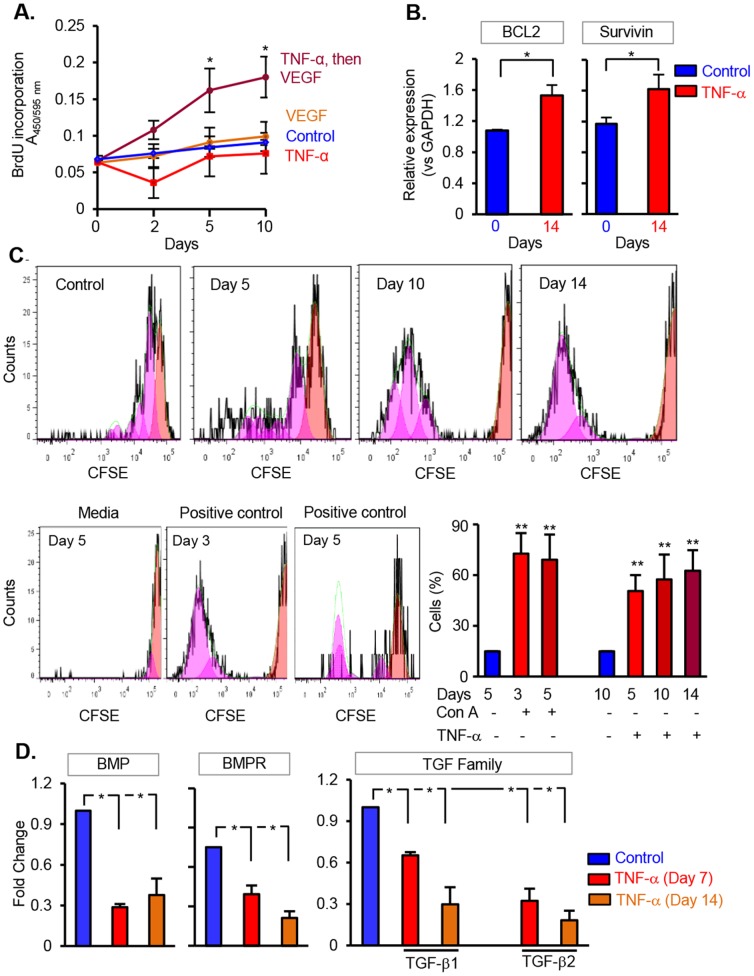
Long-term exposure to TNF-α enhances angiogenic signaling and proliferation of DPSC, *in vitro*. (**A**) BrdU incorporation assay of cells primed with TNF-α for 24 hrs and challenged with VEGF (50 ng/ml) in 3% serum containing media. Cells treated with TNF-α, VEGF, and media alone serve as control. Note an increase in the proliferation of DPSC challenged with TNF-α and VEGF at 5 and 10 days. (**B**) Real time PCR analysis of cell survival genes BCL2 and Survivin at 10 days after TNF-α treatment. (**C**) Histogram plots of CFSE fluorescence of DPSC challenged with TNF-α (primed) and VEGF for days 0, 5, 10, and 14). The plots of each day show the CFSE profiles of viable CFSE-labeled DPSC cells. The enlarged CFSE plot shows a FlowJo-generated CFSE profile, individual histograms shown subsequent to the gray filled histogram represent each cell division. The bar graph represents the percentage of cells in days 0, 5, 7, and 10 days. Concavalin A serves as a positive control. Cells cultured in media alone serve as a negative control. (**D**) Real time PCR analysis demonstrating the expression pattern of genes associated with dental-pulp longevity and mineralization. The experiments are repeated at least four times for statistical analysis. The data shown are Mean ± SD. *p<0.05.

To examine the proliferation potential of DPSC, we performed a nonisotopic BrdU incorporation assay. In order to do that, DPSC primed with TNF-α for 48 hrs were challenged with VEGF for 5 and 10 days and were labeled with BrdU (3 µg/ml) for 4 hrs at the end of the respective treatment periods. As shown in Fig. 2A, cells challenged with TNF-α (primed)+VEGF showed a significant increase in proliferation, when compared to cell treated with control, VEGF, or TNF-α alone or untreated. In parallel, qPCR analysis showed an upregulation of BCL2 and Survivin in TNF-α-treated cells on day 14 (Fig. 2B).

To further corroborate our observations, we labeled DPSC with carboxyfluorescein succinimidyl ester (CFSE) and challenged with TNF-α (primed)+VEGF to trace multiple generations of cell populations. As shown in Fig. 2C, flow cytometry analysis show that cells exposed to TNF-α for days 10 and 14 displayed an increase in the number of generations, when compared to control or day 5. These findings unambiguously explicate the role of TNF-α in mediating angiogenic proliferation and the coherent feature of DPSC response to angiogenic signaling. Cells treated with Concavalin A served as a positive control at days 3 and 5. We also determined whether TNF-α perturbs proteins essential for dental-pulp longevity and mineralization. To determine that, we performed qPCR analysis to evaluate the levels of BMP, BMPR, and TGF family of proteins in cells treated with TNF-α for 7 and 14 days. As anticipated, we observed a significant decrease in the levels of BMP, BMPR, TGF-β1 and TGF-β2 in cells exposed to TNF-α (Fig. 2D). These findings clearly suggest that prolonged exposure to proinflammatory stimuli contribute significantly to an emerging angiogenic potential of DPSC.

### Inhibition of NF-κB Signaling Restores TNF-α-Induced Angiogenic Signaling in DPSC

**Figure 3:**
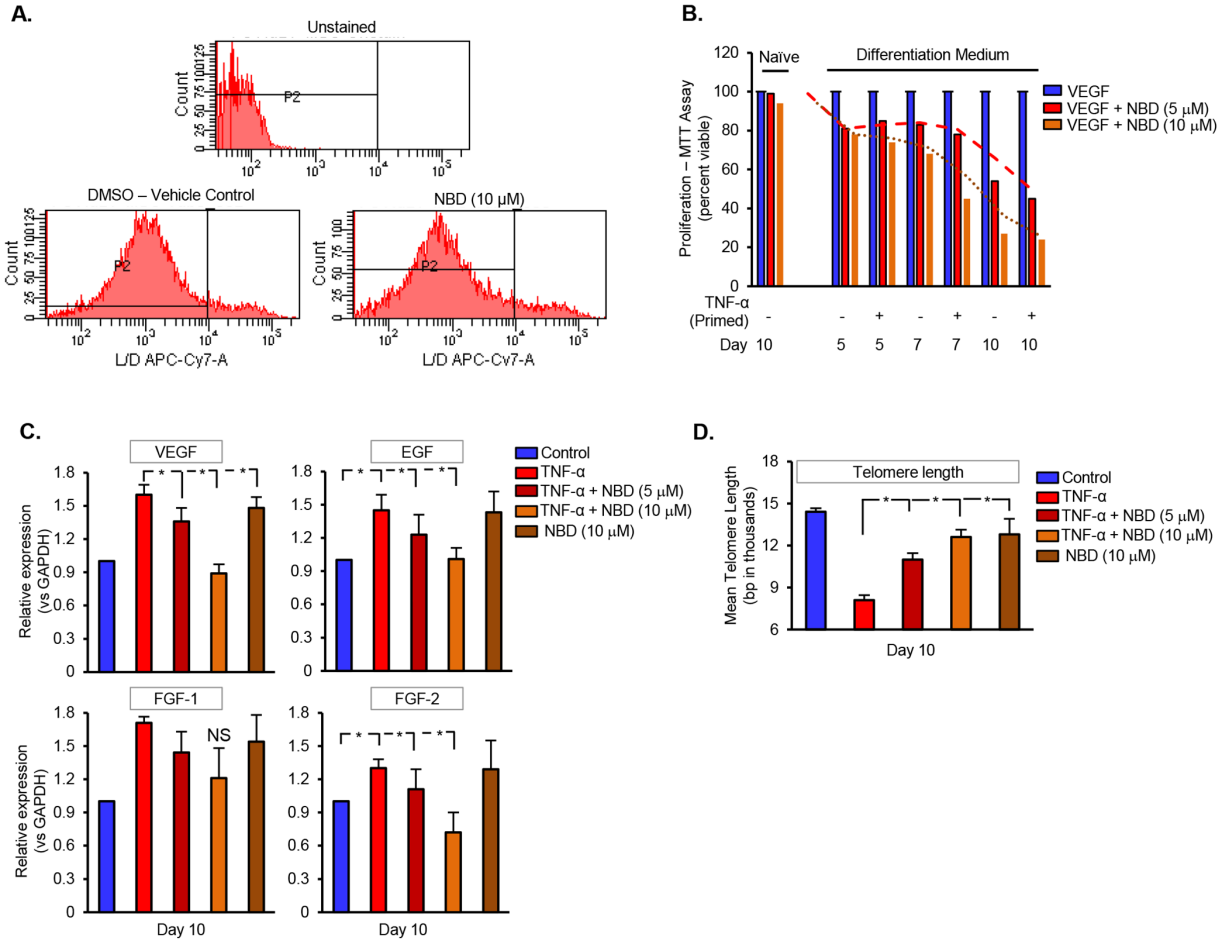
Inhibition of NF-κB signaling impedes TNF-α-induced increase in proliferation and angiogenic signaling. (**A**) Histogram plots from flow cytometry analysis show the percentage of Live/Dead (L/D) cells in the presence of vehicle control or NBD peptide. (**B**) MTT assay showing a decrease in TNF-α-induced increase in proliferation, in DPSC challenged with varying concentrations of NBD domain peptide (5 and 10 µM). (**C**) Real time PCR analysis showing the RNA expression levels of VEGF, EGF, FGF-1, and FGF-2 in DPSC treated with TNF-α in the absence or presence of NBD (5 and 10 µM). DPSC treated with NBD alone serve as a positive control. (**D**) Average telomere length was measured from total genomic DNA by using a sequence-independent multiplex qPCR technique. Data shown are the mean telomere length quantified, at day 10, in DPSC treated with TNF-α in the absence or presence of NBD. The data shown are Mean ± SD. *p<0.05 from three independent experiments. “NS” represents a non-significant difference between the test groups.

Since we observed the TNF-α-mediated increase in NF-κB expression and signaling (phosphorylation of p65 and IκB-α), we investigated the effect of NEMO-binding domain (NBD) peptide, a NF-κB signaling inhibitor on TNF-α-induced angiogenesis and phenotypic alterations in DPSC. First, we tested the effect of TNF-α or NBD peptide on the viability of DPSC using flow cytometry which combine the fluorophores APC and Cy7A [31]. Our studies exhibited no significant loss in the viability of DPSC treated with varying doses of NBD when compared to Vehicle control. DPSC unstained with APC-Cy7A serve as a negative control. In order to examine whether TNF-α-treated cells undergo changes in VEGF-induced proliferation, we treated DPSC with NBD (5 and 10 µM). Our findings show that treatment with NBD resulted in a ∼20% reduction in VEGF-induced increase in proliferation, at day 5. However, proliferation analysis performed on day 7 and day 14 showed a 40 and 80% decrease in proliferation, respectively (Fig. 3B). These findings suggest that NF-κB inhibition restored TNF-α-induced increase in DPSC proliferation. Furthermore, to determine whether NF-κB inhibition reinstated the angiogenic signaling, we examined the expression levels of VEGF, EGF, and FGF family of growth factors using qPCR analysis. DPSC treated with TNF-α in combination with NBD (10 µM) significantly decreased or restored the levels of growth factors. However, lower dose (5 µM) showed no significant changes.

It is fairly evident from our studies that prolonged exposure to TNF-α may emerge DPSC in to an apoptotic-resistant phenotype, a condition in which dysregulation of telomere binding proteins occurs leading to telomere shortening. Therefore, we urged to determine whether prolonged exposure of TNF-α influenced telomere shortening. In order to do that, DPSC treated with TNF-α for 14 days in the presence or absence of NBD were used for sequence-independent multiplex qPCR analysis. It is interesting to note from the observations that cells treated with TNF-α for 14 days exhibited a significant decrease in telomere length, which was eventually restored when treated with NBD peptide (5 and 10 µM) (Fig. 3C). These findings further corroborate our hypothesis that TNF-α-induced initial apoptosis emerges DPSC in to an angiogenic phenotype.

### Early Inhibition of NF-κB Potentiates DPSC Mineralization and Differentiation

**Figure 4:**
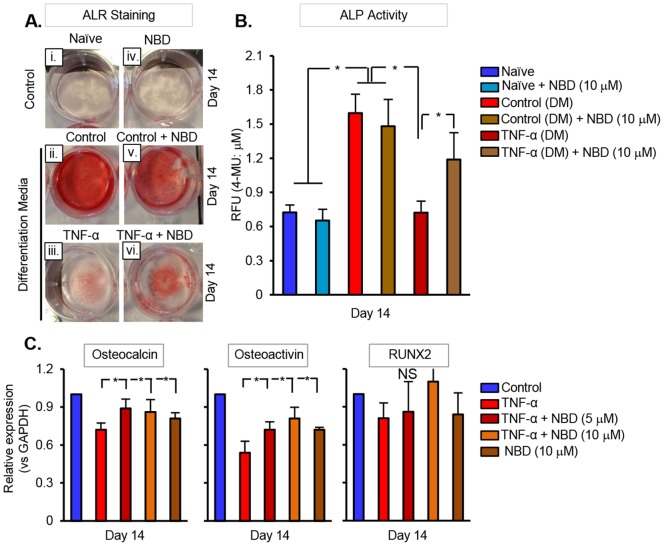
NF-κB inhibition ameliorates TNF-α-induced changes in mineralization-associated proteins and mineralized nodule formation. (**A**) Mineralized nodule formation was assessed by ALR staining. DPSC challenged with TNF-α and VEGF in the absence or presence of NBD was visualized for ALR staining at day 14. NBD was replaced every 4 days (at least 3 times during the mineralization process). (**B**) Alkaline phosphatase enzyme activity was quantified in DPSC treated with TNF-α and VEGF, in the absence or presence of NBD. (**C**) Real time PCR analysis showing the expression levels of the differentiation markers; osteocalcin, osteoactivin, and Runx2 in cells challenged with TNF-α and VEGF, in the absence or presence of NBD. The data shown are Mean ± SD. *p<0.05, from at least three independent experiments. “NS” represents a non-significant difference between the test groups.

We investigated whether prolonged exposure of TNF-α impedes odontogenesis in DPSC. In order to do that, DPSC cultured in odonto-inductive medium were challenged with TNF-α and VEGF, and were subjected to alizarin red staining. Compared with untreated DPSC, the number of mineralized nodules was significantly increased in odonto-inductive medium; however, when the cells were treated with TNF-α the number of nodules diminished, significantly (Fig. 4A; *i*–*iii*). This indicates that prolonged exposure to TNF-α impedes the mineralization potential of DPSC. The effect of short-term TNF-α treatment on mineralization is merely feasible to examine, as it transpires 2–3 weeks after culture in odonto-inductive medium.

To identify the involvement of NF-κB signaling, DPSC cultured in odonto-inductive medium in the presence of NBD (10 µM) for 14 days were subjected to ALR staining. Interestingly, we observed a marked increase in the appearance of mineralization nodules in cells co-treated with NBD (Fig. 4A: *vi*), when compared to cells treated with NBD in the absence of TNF-α (Fig. 4A: *v*) or control (Fig. 4A: i*v*). ALP enzyme activity assay also revealed a significant restoration of the differentiation potential when treated with NBD, as shown in Fig. 4B. To investigate whether inhibition of NF-κB induces the expression of genes associated with mineralization, the expression of osteocalcin, osteoactivin, and RUNX2 mRNA were determined in the absence or presence of NBD domain peptide. PCR analysis showed a significant decrease in the expressions of osteocalcin, osteoactivin, and RUNX2 mRNA in TNF-α-treated DPSC (Fig. 4C). Nevertheless, cells treated with the combinations of VEGF (primed)+TNF-α and NBD peptide showed a significant increase in the expressions of osteocalcin and osteoactivin, when compared to TNF-α or NBD peptide treatment alone. RUNX2 showed a trend to increase in its level; however, it failed to show significance.

## Discussion

During inflammatory episodes, dental pulp is more sensitive to changes in tissue pressure and requires an active drainage system to eliminate excess fluid and macromolecular substances. This effective system plays a crucial role in the repair and wound healing processes. In particular, the enhanced cellular differentiation and neovascularization of the dental pulp is an important event for pulp healing [30]. The pulpal healing potential is associated with the ability of dental pulp cells to secrete growth factors, including angiogenic factors [25], [28]. Various reports have demonstrated the role of inflammatory cytokines on dental pulp longevity and differentiation potential [32]. However, this has been a matter of continuing controversy because inflammatory stimulus is always paired with the angiogenic signaling. Therefore, in our studies we investigated the effect of TNF-α in combination with VEGF on DPSC proliferation and differentiation. In the present study, we demonstrated that TNF-α along with VEGF increased the proliferation of DPSC with a concomitant decrease in telomere length.

TNF-α was shown to activate NF-κB, a master transcription factor that regulates a variety of pro-inflammatory cytokines and angiogenic factors in dental pulp fibroblasts and oral epithelial cells [33]–[36]. However, the mechanisms by which NF-κB signaling might be responsible for the angiogenic signaling and the resultant effects on its differentiation potential of DPSC remain unknown. In this study, we found that TNF-α treatment significantly induced p65 phosphorylation, a transactivation domain critical for NF-κB transcription [18]. In parallel, we also observed an increase in the phosphorylation of IκB-α, a process essential for NF-κB activation [18], [22]. To examine whether TNF-α-induced NF-κB signaling contributes to the angiogenic proliferation and differentiation, we blocked NF-κB using NBD. It was observed that nearly 40% to 50% of the increase in TNF-α/VEGF-induced proliferation was significantly decreased in the DPSC population treated with NBD. Additionally, it is interesting to note from our findings that TNF-α-induced abrogation of DPSC mineralization and differentiation was significantly ameliorated in cells treated with NBD peptide.

Our findings also reveal an intriguing possibility that chronic exposure to TNF-α leads to a hyperproliferative phenotype of DPSC with a simultaneous increase in the angiogenic signaling with no significant alterations in the increase of cell surface markers prevailing to the differentiation of DPSC into cells of endothelial lineage (Figure S1: A and B). Furthermore, as shown in Figure S1: A and B; panels *iv* and *vi*, we observed additional population (CD31^−^) of cells positive to CD105 and CD29. These findings instigated us to investigate the existing population of cells with the characteristics similar to DPSC.

**Figure 5:**
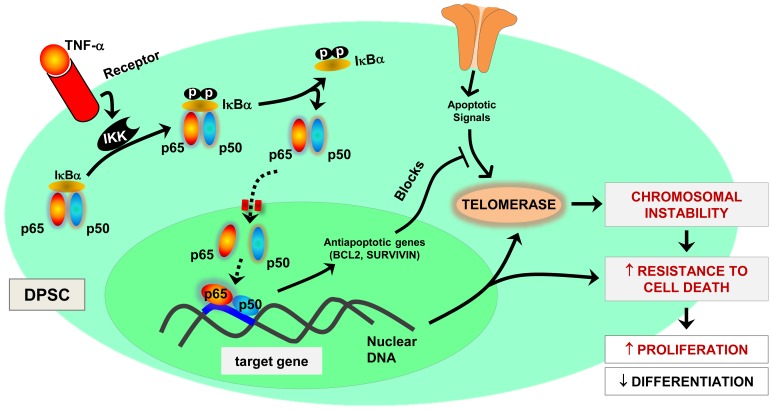
Signaling pathway downstream of inflammatory mediator (TNF-α) signaling in human DPSC. Prolonged exposure to TNF-α induce NF-κB signaling axis, which in turn induce an enhanced antiapoptotic gene expression and decreased telomere length. The resultant chromosomal instability leads to an increase in the proliferation of DPSC, with an impaired differentiation potential.

In recent years, studies have identified a unique population of cells termed CD31^−^ Side Population (SP) [37] from pulp tissue with a higher regenerative potential in the ischemic disease models and the pulp regeneration model [38], [39]. SP fraction from permanent teeth was shown to be increased to approximately 5% upon stimulation with ischemic culture [39]. Therefore, our studies elucidated whether the additional staining population observed in TNF-α treated cells (Figure S1; *iv* and *vi*) were the SP cells. In order to address this, we performed flow cytometry analysis probing for ATP-binding cassette (ABC-G2) – an important determinant of the SP phenotype [39], [40]. It is interesting to note from our findings that DPSC challenged with TNF-α showed an increased surface-level expression of ABC-G2 (9.6%±1.86%) when compared to control (0.46%±0.12%) (Figure S1: C). These results are in accordance with the earlier findings [40] that SP fraction of cells potentiates during inflammatory *mileu*. However, the role or contribution of SP cells in pulp regeneration remains unclear. Further studies are warranted to elucidate the synergistic effect of SP cells in dental pulp. In conclusion, our results are the first to demonstrate (Fig. 5) that TNF-α-induced NF-κB signaling and the ensuing upregulation of antiapoptotic signaling contribute significantly to the enhanced proliferation of DPSC, while impairing its differentiation potential.

## Supporting Information

Figure S1 Surface molecule characterisation of human DPSC in the absence or presence of TNF-α. (A) Flow cytometric analysis of cultured DPSC in the absence or presence of TNF-α at day 10 revealed the non-significant difference in the expression of CD29^+^, CD105^+^, and CD31^+^ in the population negative for CD45 (CD45^−^). (B) The bar diagram demonstrates the percentage change in the number of events. The data shown are Mean ± SD. *p<0.05, from at least four independent experiments. “NS” represents a non-significant difference between the test groups. (C) ABC-G2 expression in DPSC either challenged without or with TNF-α at day 10. TNF-α treated cells show ABC-G2^+^ (9.57%±1.86%) cells when compared to control (0.46%±0.12%).
(TIF)
