# Supplementary figures and images for: Chronic Inflammation and Angiogenic Signaling Axis Impairs Differentiation of Dental-Pulp Stem Cells
Source: PLoS One. 2014 Nov 26;9(11):e113419. doi: 10.1371/journal.pone.0113419 (PMC4245135; doi:10.1371/journal.pone.0113419)

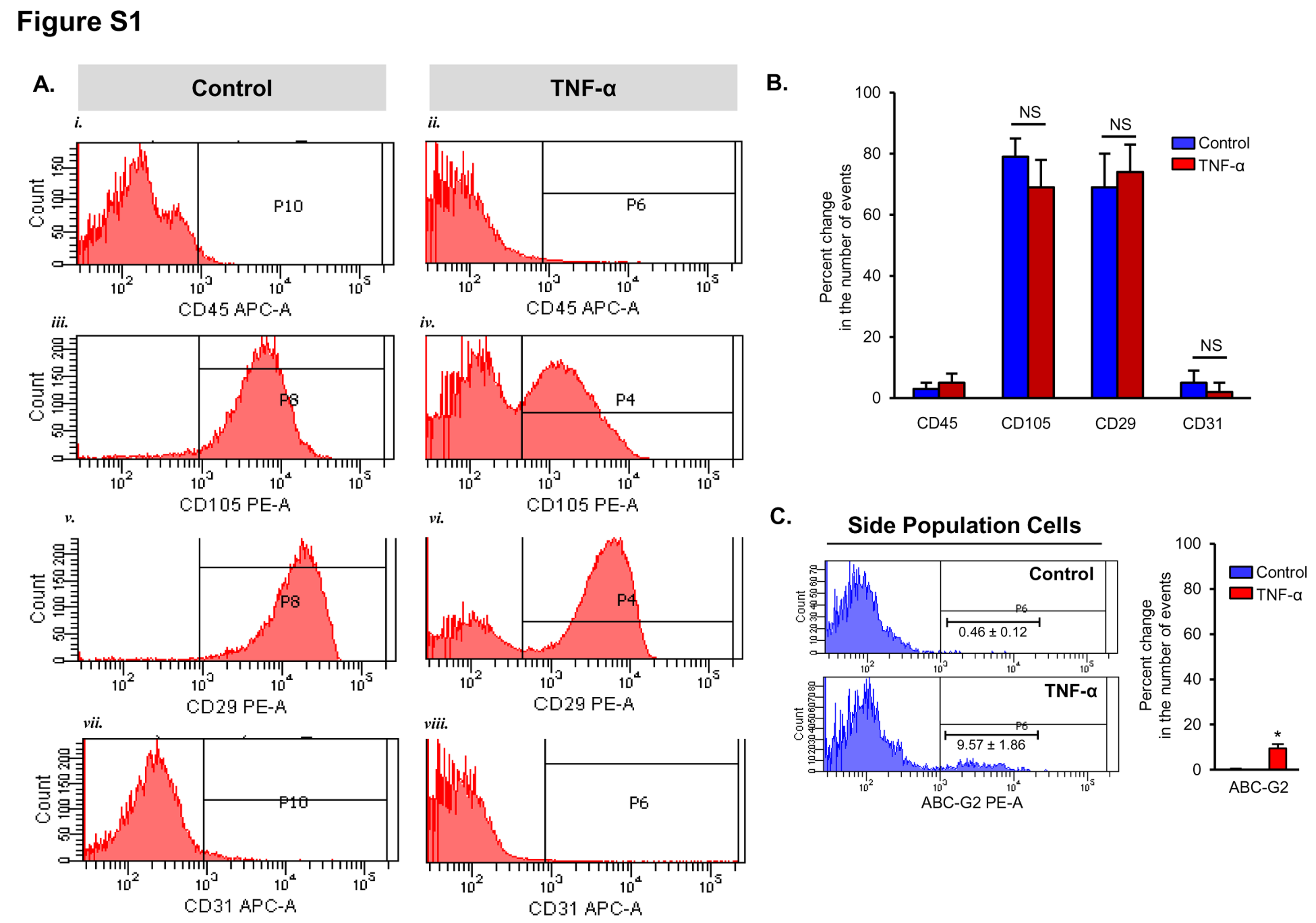

Supplement: Figure S1 — Surface molecule characterisation of human DPSC in the absence or presence of TNF-α. (A) Flow cytometric analysis of cultured DPSC in the absence or presence of TNF-α at day 10 revealed the non-significant difference in the expression of CD29+, CD105+, and CD31+ in the population negative for CD45 (CD45−). (B) The bar diagram demonstrates the percentage change in the number of events. The data shown are Mean ± SD. *p<0.05, from at least four independent experiments. “NS” represents a non-significant difference between the test groups. (C) ABC-G2 expression in DPSC either challenged without or with TNF-α at day 10. TNF-α treated cells show ABC-G2+ (9.57%±1.86%) cells when compared to control (0.46%±0.12%). (TIF) [file pone.0113419.s001.tif]
